# Genes for the high life: New genetic variants point to positive selection for high altitude hypoxia in Tibetans

**DOI:** 10.24272/j.issn.2095-8137.2017.031

**Published:** 2017-05-18

**Authors:** Nina G. Jablonski

**Affiliations:** The Pennsylvania State University, University Park, PA 16902, USA

People living on the high plateaus of the world have long fascinated biological anthropologists and geneticists because they live in "thin air" and epitomize an extreme of human biological adaptation. Far from posing obstacles to human habitation, the hypoxic conditions of the Tibetan Plateau, the Andean highlands, and the Amhara Plateau of Ethiopia have served as the contexts for natural experiments in human adaptability (Beall, 2014). To the studies of the high-altitude human phenotype of the latter half of the twentieth century have been added studies of the complementary high-altitude genotype in the first decades of the twenty first century. Studies of genetic adaptation in Tibetans have commanded great interest because humans there live at altitudes around 4 000 m above sea level, breathe air that has an oxygen concentration about 40% lower and experience ultraviolet radiation about 30% higher than at sea level. An extreme habitat indeed.

Among the most dramatic adaptations to hypobaric hypoxia seen in high-altitude-dwelling Tibetans are the higher levels of the vasodilator, nitric oxide, in their exhaled breath and blood. Regulation of the pulmonary vascular response to hypoxia has been recognized as a key feature of the high altitude adaptation of Tibetans, but until recently little was known of the genetic basis of this phenomenon. Two recent papers in this journal from the laboratory of Su Bing, by Guo et al. (2017) and Zheng et al. (2017), now shed light on this. These papers reveal that the *GCH1* (GTPcyclohydrolase Ⅰ) gene and the *EP300* (histone acetyltransferase p300) gene, which are both involved in maintaining nitric oxide synthetase (NOS) function and normal blood pressure, harbor potentially adaptive variants in Tibetans. Both genes show high allelic divergence between Tibetan and lowland-dwelling Han Chinese, with signals of positive selection in the Tibetans.

These discoveries add to the extensive catalogue of evidence indicating that the Tibetan genome has undergone natural selection at multiple loci concerned with distinct aspects of blood-related phenotypes, including regulation of hemoglobin phenotype (Beall, 2014) and folate metabolism (Yang et al., 2017). Humans have been traversing the Tibetan Plateau for more than 30 000 years and have been permanent residents there for at least 6 000 years (Qiu, 2015). The true evolutionary significance of the unique genetic variants carried by Tibetans still remains to be established through "ground truthing" of human survival and reproduction (Beall, 2014), but this new genetic information provides further important and suggestive evidence that natural selection along with culture shaped humanity's adaptations to some of the planet's most extreme environments. 
